# Frame-Insensitive Expression Cloning of Fluorescent Protein from *Scolionema suvaense*

**DOI:** 10.3390/ijms19020371

**Published:** 2018-01-26

**Authors:** Yuki Horiuchi, Danai Laskaratou, Michel Sliwa, Cyril Ruckebusch, Kuniyuki Hatori, Hideaki Mizuno, Jun-ichi Hotta

**Affiliations:** 1 Department of Bioengineering, Graduate School of Science and Engineering, Yamagata University, 992-8510 Yonezawa, Japan; tts99002@st.yamagata-u.ac.jp; 2 Biomolecular Network Dynamics, Biochemistry, Molecular and Structural Biology Section, KU Leuven, Celestijnenlaan 200g Box 2403, 3001 Leuven, Belgium; danai.laskaratou@kuleuven.be (D.L.); hideaki.mizuno@kuleuven.be (H.M.); 3 Laboratoire de Spectrochimie Infrarouge et Raman, Université de Lille, CNRS, UMR 8516, LASIR, F59 000 Lille, France; michel.sliwa@univ-lille1.fr (M.S.); cyril.ruckebusch@univ-lille1.fr (C.R.); 4 Department of Bio-System Engineering, Graduate School of Science and Engineering, Yamagata University, 992-8510 Yonezawa, Japan; khatori@yz.yamagata-u.ac.jp

**Keywords:** fluorescent protein, expression cloning, reading frame, frameshift

## Abstract

Expression cloning from cDNA is an important technique for acquiring genes encoding novel fluorescent proteins. However, the probability of in-frame cDNA insertion following the first start codon of the vector is normally only 1/3, which is a cause of low cloning efficiency. To overcome this issue, we developed a new expression plasmid vector, pRSET-TriEX, in which transcriptional slippage was induced by introducing a DNA sequence of (dT)_14_ next to the first start codon of pRSET. The effectiveness of frame-insensitive cloning was validated by inserting the gene encoding eGFP with all three possible frames to the vector. After transformation with one of these plasmids, *E. coli* cells expressed eGFP with no significant difference in the expression level. The pRSET-TriEX vector was then used for expression cloning of a novel fluorescent protein from *Scolionema suvaense*. We screened 3658 *E. coli* colonies transformed with pRSET-TriEX containing *Scolionema suvaense* cDNA, and found one colony expressing a novel green fluorescent protein, ScSuFP. The highest score in protein sequence similarity was 42% with the chain c of multi-domain green fluorescent protein like protein “ember” from Anthoathecata sp. Variations in the N- and/or C-terminal sequence of ScSuFP compared to other fluorescent proteins indicate that the expression cloning, rather than the sequence similarity-based methods, was crucial for acquiring the gene encoding ScSuFP. The absorption maximum was at 498 nm, with an extinction efficiency of 1.17 × 10^5^ M^−1^·cm^−1^. The emission maximum was at 511 nm and the fluorescence quantum yield was determined to be 0.6. Pseudo-native gel electrophoresis showed that the protein forms obligatory homodimers.

## 1. Introduction

Fluorescent proteins have become a valuable tool as a reporter system for visualizing detailed structure of cells since the first demonstration of green fluorescent protein (GFP) expression and observation with fluorescence microscopy in *Caenorhabditis elegans* [1,2]. Recent progress in the development of fluorescent proteins and fluorescence microscopy realizes even nanometer resolution by means of super resolution fluorescence microscopy [3,4,5,6,7]. The technique relies on the ON/OFF blinking properties [8] found in many fluorescent proteins screened from wild creatures, or mutated from existing ones [9,10]. Nowadays, efficient rational design of mutants using the knowledge of protein structure has become feasible [11,12]. However, cloning from wild creatures is still essential to acquire novel fluorescent proteins [13]. There are several ways to clone a fluorescent protein gene from cDNA pools synthetized from mRNA. The most efficient way is polymerase chain reaction (PCR), using primers designed based on the similarity of amino acid sequence with known fluorescent proteins [14,15,16]. This method, though, is not applicable to extracting a low sequence similarity gene from a cDNA pool. Expression cloning is an alternative way to find a gene encoding fluorescent protein(s) with unknown sequences from a cDNA pool. The entire pool of cDNA from mRNA is introduced to expression vectors and screened by searching for fluorescent colonies. There is a large variety of vectors applicable to expression cloning. Among them, pRSET is designed for efficient protein expression in bacterial cells by making use of T7 phage promoter and the T7 phage gene 10 leader. The gene 10 leader serves as a ribosome-binding site on mRNA, which enhances protein expression. Since the T7 phage gene 10 leader sequence needs to be placed after the start codon, the gene encoding a target protein should be inserted in-frame after the leader sequence. The reading frame of the respective gene is stochastic when a cDNA library is constructed on the pRSET vector. Hence, proper protein expression is expected only in 1/3 of the clones. This is one of the bottlenecks of the expression cloning. Researchers are often limited in starting material; they have to use a fragment of the specimen collected in the field or need to store RNA in preserving solution. Therefore, the library size and the amount of materials are limited. In such cases, frame-insensitive expression cloning can increase the chance of finding a rare expressing gene, in our case a fluorescent protein gene.

**Figure 1:**
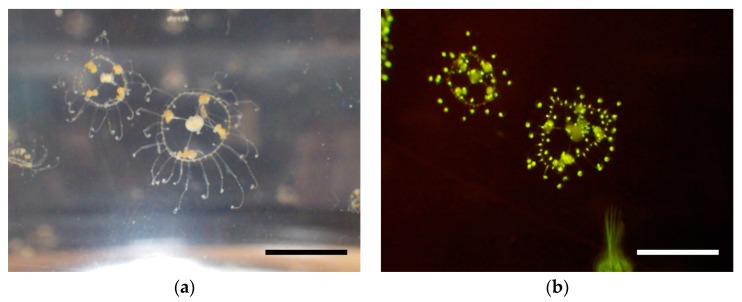
A jellyfish *Scolionema suvaense*. (**a**) Image under ambient light. (**b**) Fluorescence image under blue LED (470 nm) illumination. Green fluorescence was observed through an orange transparent acrylic plate (A300, Hikari Co., Ltd., (Osaka, Japan). Scale bars are 10 mm.

It is known that poly dA or poly dT sequences often induce slippage of RNA polymerase during transcription initiation, which results in production of mRNA with frame-shift [17,18,19]. This phenomenon, so-called transcriptional slippage, has been incorporated into λ expression vectors; frame insensitive phage vectors λTriplEx and λTriplEx2 (Clontech Laboratories, Inc., Mountain View, CA, USA) are commercially available. In this study, we introduced the transcriptional slippage into a plasmid-based expression vector for frame-insensitive expression cloning. In this system, mRNAs with all three reading frames are expected to be transcribed from one plasmid, achieving 3 times higher cloning efficiency. By using this system, we performed expression cloning of fluorescent protein from a jellyfish *Scolionema suvaense* (Figure 1), and successfully got a novel green fluorescent protein, whose highest score in sequence similarity to already existing fluorescent proteins was only 42%.

## 2. Results

**Figure 2:**
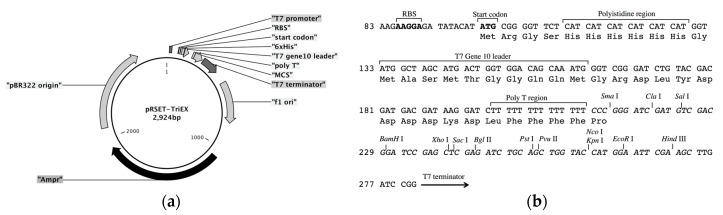
Structure of frame-insensitive bacterial expression cloning vector, pRSET-TriEX. (**a**) Vector map of pRSET-TriEX. (**b**) Detailed information around multiple cloning site (MCS). The DNA sequence (dT)_14_ and restriction enzyme sites (SmaI, ClaI, and SalI) are inserted after original start codon of pRSET B.

**Figure 3:**
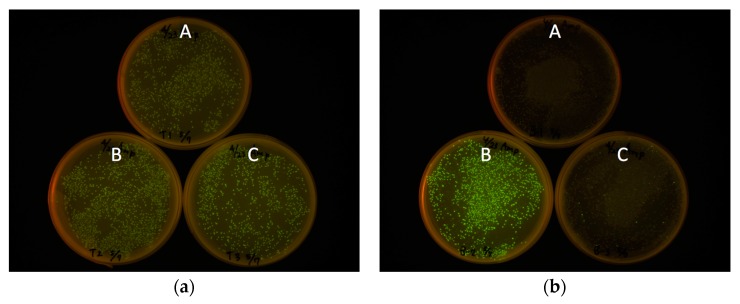
Frame-insensitive expression of eGFP with pRSET-TriEX vector. (**a**) The genes encoding eGFP with different reading frames were inserted in pRSET-TriEX. (**b**) pRSET B was used as a negative control. The labels A, B, and C indicate the frames of eGFP CDS.

The frame-insensitive expression cloning vector pRSET-TriEX was made by inserting the DNA sequence for the transcriptional slip (dT)_14_ together with restriction enzyme sites (SmaI, ClaI, and SalI) into the BamHI site of the original vector pRSET B (Figure 2). To evaluate the effect of the transcriptional slip, the gene encoding eGFP with A, B, or C frame was inserted into pRSET-TriEX and subjected to *E. coli* JM109(DE3) transformation, using the insertion to pRSET B as a negative control (Figure 3). For all constructs, the start codon of eGFP coding DNA sequence (CDS) was removed to avoid dual initiation. All colonies are moderately fluorescent for the bacteria transformed with pRSET-TriEX backbone, regardless of the reading frame. This stands in striking contrast to the transformation with the pRSET B backbone; colonies of transformants with the frame B plasmid showed strong fluorescence, whereas transformants with the frame A or C plasmid were nonfluorescent, except for a small number of colonies which might be due to leftover frame B fragments from the PCR template (see Methods for details). We concluded that frame-insensitive expression with transcriptional slip worked with the plasmid-based bacterial expression vector system. 

**Figure 4:**
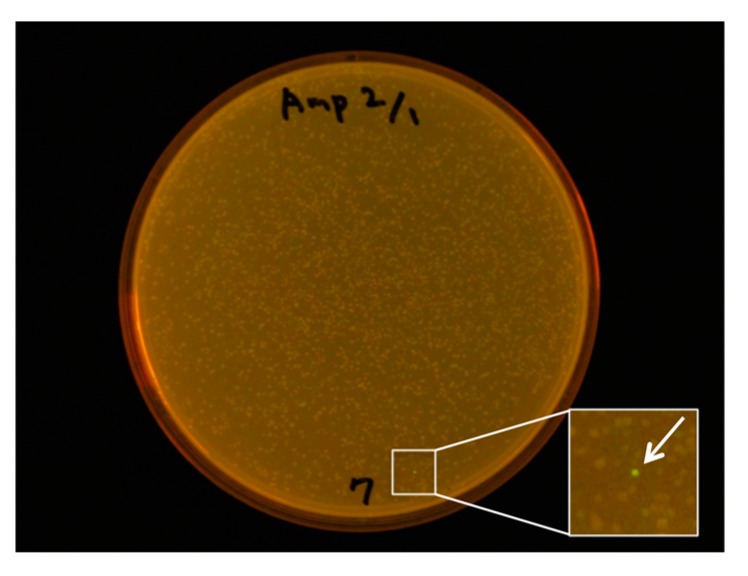
Expression cloning of fluorescent protein from *Scolionema suvaense* with the frame-insensitive pRSET-TriEX vector. Screening of 3658 colonies in total was performed and one green fluorescent colony was found only on the plate, shown here in the bottom right corner.

With this frame-insensitive vector, we attempted expression cloning of a novel fluorescent protein from cDNA pools synthesized from mRNA of *Scolionema suvaense* (Figure 4). Screening of 3658 colonies resulted in finding one fluorescent colony. The plasmid in this colony was amplified, prepared, and subjected to DNA sequencing with the T7 promoter and T7 terminator sequencing primers. The length of the CDS was 690 bp with a theoretical molecular weight of 26.0 kDa. This fluorescent protein was named ScSuFP.

**Figure 5:**
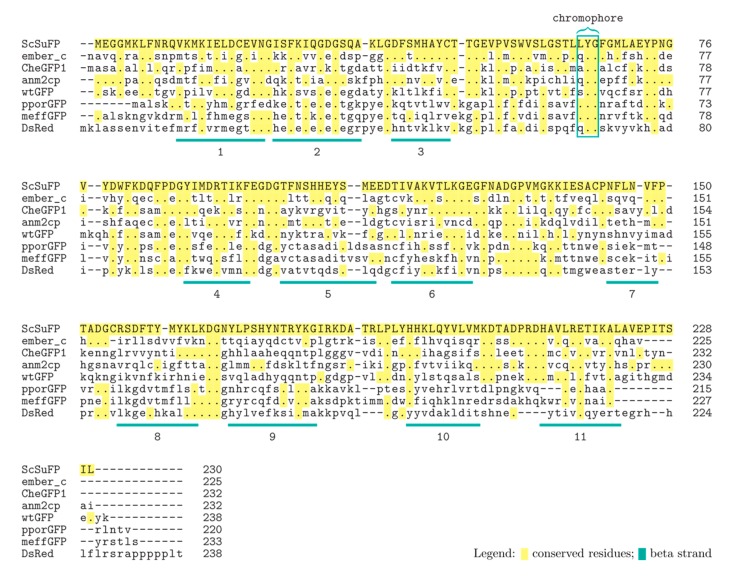
Alignment of the amino acid sequence of ScSuFP with ember_c, CheGFP1, anm2cp, wtGFP, pproGFP, meffGFP and DsRed. Identical amino acids are indicated with dots and highlighted with yellow color. Non-conserved residues are shown in lowercase and have no highlighting. The positions of chromophores are enclosed in a box. Beta-strands are underlined and numbered based on the wtGFP sequence. The alignment was made using Clustal Omega (Available online: https://www.ebi.ac.uk/Tools/msa/clustalo/) and the highlighting/labeling with TEXshade (Available online: https://www.uni-kiel.de/pharmazie/chem/Prof_Beitz/texshade.html) [24,25,26].

BLAST search [20] was applied to find fluorescent proteins that show high sequence similarity with ScSuFP: three proteins with the highest similarity were ember_c, CheGFP1, and anm2cp with identity percentage of 42%, 39%, and 36%, respectively [15,21,22]. All three are proteins isolated from Hydrozoa, same as *Scolionema suvaense*. None of Anthozoa fluorescent proteins showed high sequence similarity. The amino acid sequence of ScSuFP was aligned with hydrozoan fluorescent proteins showing highest sequence similarity (Figure 5) [23]. Alignments with wtGFP and DsRed are also shown as representatives of Hydrozoa and Anthozoa fluorescent proteins, respectively, since these are predecessors of numerous fluorescent protein mutants commonly used nowadays. The chromophore of ScSuFP is composed of leucine-tyrosine-glycine (LYG), which is unique among naturally occurring Hydrozoa fluorescent proteins. So far, two Anthozoa fluorescent proteins, pporGFP and meffGFP, have been reported to have the LYG chromophore. The ScSuFP sequence was also aligned to these proteins. The N- and C-terminal sequences of ScSuFP vary from other fluorescent proteins, except for the N-terminus of anm2cp. None of the β-strands of the Anthozoa fluorescent proteins shown here showed significant similarity to ScSuFP. In comparison to Hydrozoa fluorescent proteins aligned here, 1st, 4th, and 6th β-strands of ScSuFP showed relatively high similarity, whereas 7th, 8th, 9th, and 10th β-strands were almost completely different. When comparing with wtGFP, the 1st β-strand has 6 out of 12 amino acid residues in common, the 4th β-strand has 5 out of 9, and the 6th β-strand has 5 out of 11.

**Figure 6:**
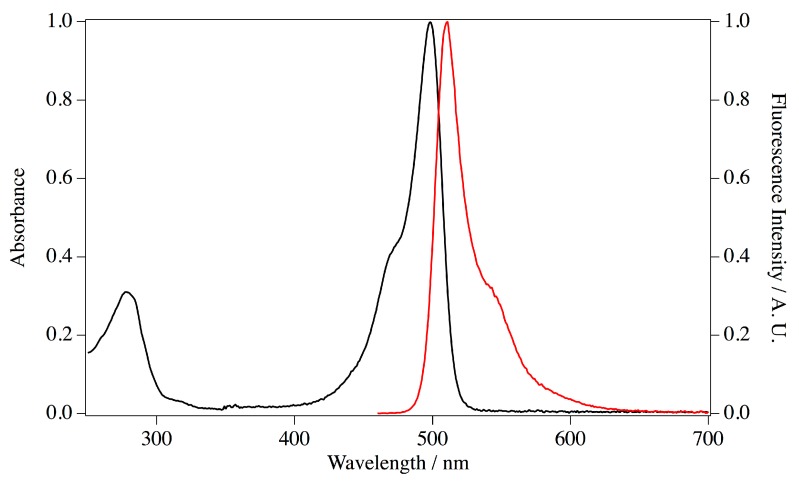
Absorption (black line) and fluorescence emission (excitation at 450 nm, red line) spectrum of ScSuFP at pH 7.0. Absorption maximum is at 498 nm and emission maximum is at 511 nm. Absorbance and fluorescence intensity are normalized at 498 nm and 511 nm, respectively.

Next, the CDS of ScSuFP was extracted by PCR amplification and subcloned into an empty pRSET B vector. Recombinant ScSuFP was expressed in *E. coli* transformants with this plasmid and purified by metal chelating column chromatography. Imidazole in the eluate was removed by pathing through a desalting column. The purified protein appeared as a single band on SDS PAGE at the estimated size of 30 kDa. Absorption and fluorescence spectra were measured for the purified ScSuFP (Figure 6). The absorption band at 280 nm was assigned to amino acid residues and a structured visible band was assigned to the chromophore. The absorption maximum of the visible band was at 498 nm and the molar extinction coefficient was 1.17 × 10^5^ M^−1^·cm^−1^ [27,28]. The protein concentration to estimate the extinction coefficient was determined by the alkaline-denatured method, which reflects the concentration of matured chromophore. The concentration was >90% of the total ScSuFP determined with the theoretical extinction coefficient at 280 nm, which was calculated based on the amino acid sequence, suggesting a good folding of ScSuFP in the bacteria. The emission spectrum has a maximum at 511 nm and the fluorescence quantum yield was calculated to be 0.6 using the comparative method.

**Figure 7:**
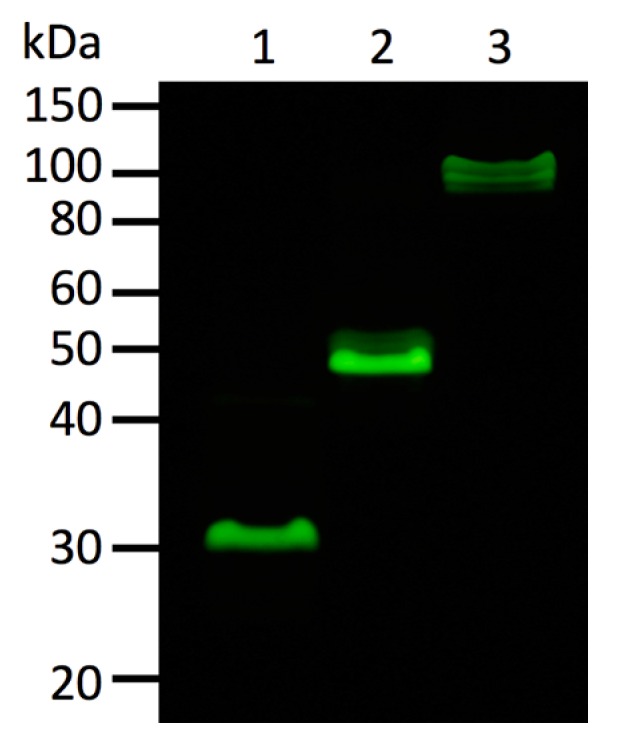
Dimer formation of ScSuFP in solution revealed by pseudo-native gel-electrophoresis. eGFP and Kaede were used as a control of monomer and obligatory tetramer, respectively. The photograph was taken through ALSC-56 filter under blue light illumination at 480 nm. Molecular weight standards are shown on the left of the gel. Lanes: 1—eGFP (monomer); 2—ScSuFP; 3—Kaede (tetramer).

Oligomeric state of fluorescent proteins was examined with pseudo-native gel-electrophoresis (Figure 7). eGFP and Kaede were used as oligomeric fluorescent protein standard of monomer and tetramer, respectively. ScSuFP was seen between eGFP and Kaede with molecular weight ~50 kDa, which shows that ScSuFP was in a dimeric state in solution.

## 3. Discussion

By introducing a (dT)_14_ sequence for transcriptional slip, we could successfully produce a frame-insensitive bacterial expression plasmid vector. With the transcriptional slip, mRNA with three different reading frames is produced. On our system, the fraction of mRNA in respective reading frame was estimated to roughly 1/3, since a similar level of expression was observed in all transformants with the gene encoding eGFP with different frames. The expression level was lower than in the case of in-frame construction in a conventional plasmid vector (pRSET), but the expression level with the frame-insensitive plasmid was enough for expression screening of fluorescent proteins. By applying this method, we successfully isolated the florescent protein from *Scolionema suvaense*. Sequencing data showed that the CDS of the single fluorescent colony identified during screening of ScSuFP was in frame with the original start codon. However, if we use conventional expression vectors, in theory we need 3 times of colonies (~10,000). Frame-insensitive expression cloning will give three times higher cloning efficiency for fluorescent protein genes.

Due to the minimum sequence similarity of ScSuFP with any of reported fluorescent proteins, especially the deviating N- and/or C-terminal sequences, it would have been impossible to get the ScSuFP gene with a cloning method based on sequence similarity, such as PCR. The discovery of ScSuFP establishes the importance of expression cloning. The frame-insensitive bacterial expression system is expected to serve as an effective tool to find novel fluorescent proteins with unique sequences. 

The LYG chromophore is also of particular interest. A search in the fluorescent protein database of osFP reveals that the LYG chromophore is quite uncommon among fluorescent proteins [29]. In fact, only 5 out of the 418 entries of the database shared this chromophore sequence: GFP (S65L), mTagBFP [30], mGeos-L [31], meffGFP, and pporGFP. Among those five fluorescent proteins, only meffGFP and pporGFP are naturally occurring [22], while the remaining three are the result of rational mutagenesis. Interestingly, both meffGFP and pporGFP belong to Anthozoa species and did not show high sequence similarity to ScSuFP. So far, no other Hydrozoa fluorescent proteins with LYG chromphores have been reported.

Spectroscopic analysis revealed that ScSuFP has a molar extinction coefficient of 1.17 × 10^5^ M^−1^·cm^−1^, which is quite a high value for a naturally occurring green fluorescent protein. The chromophore is placed close to the β-strands 7, 8, and 10. Generally, side chains on these strands facing the inside of the β-barrel have a strong impact to the photophysical properties. These β-strands of ScSuFP were quite unique compared to other fluorescent proteins, whereas the other side of the β-barrel (around β-strands 1, 4, and 6) was rather conserved. In addition, the β-barrel close to the chromophore is expected to be the interface of the ScSuFP homodimer. The unique arrangements of side chains at this part of the β-barrel, as well as the dimer formation, might help folding of the fluorescent protein. Details of the structural properties of ScSuFP remain to be explored.

## 4. Materials and Methods

A jellyfish *Scolionema suvaense* from the Japanese seacoast was used as a source of mRNA extraction. *Scolionema suvaense* was cultured for a few days in an aquarium tank with nauplius of *Artemia salina* as food, and extraction of the mRNA was performed. The typical temperature of the habitat of *Scolionema suvaense* was 20–23 °C [32].

Expression vector pRSET-TriEX was constructed as follows. Forward and reverse DNA oligomers that contain (dT)_14_ were synthesized by Integrated DNA Technologies (IDT). The forward oligomer BamHI-TriEX_f (GAT CTT TTT TTT TTT TTT CCC GGG ATC GAT GTC GAC G) and reverse oligomer BamHI-TriEX_r (GAT CCG TCG ACA TCG ATC CCG GGA AAA AAA AAA AAA A) were annealed to form double-stranded DNA fragments, which have sticky ends to BamHI sites. The 5′ ends of the double-stranded DNA fragments were phosphorylated with T4 Polynucleotide Kinase (M0201S, New England Biolabs Inc., Ipswich, MA, USA). pRSET B was digested with BamHI, and 5′ phosphate groups were removed using Antarctic Phosphatase (M0289S, New England Biolabs Inc.). The DNA fragments containing (dT)_14_ sequences were inserted into the BamHI site using DNA Ligation Kit Version 1.0 (Takara Bio Inc., Kusatsu, Japan). *E. coli* competent cells (JM109(DE3), Promega Corporation, Madison, WI, USA) were transformed with the constructed vector. Miniprep was performed with 2 mL of culture in a Luria Bertani (LB) medium supplemented with 100 µg/mL ampicillin (Amp), and the sequence was checked to ensure the vector is correctly constructed.

Expression level was visually inspected in terms of brightness from colonies expressing eGFP. The eGFP gene with three frames after the BamHI site was amplified by PCR. Three forward primers with different reading frames—BamHI-eGFP-F (CGG GAT CCG TGA GCA AGG GCG AGG AG), BamHI-C-eGFP-F (CGG GAT CCC GTG AGC AAG GGC GAG GAG), and BamHI-CC-eGFP-F (CGG GAT CCC CGT GAG CAA GGG CGA GGA G)—together with an identical reverse primer—EcoRI-eGFP-R (CCG GAA TTC TTA CTT GTA CAG CTC GTC CAT GC)—and the template eGFP/pREST were used for PCR to amplify the gene encoding eGFP with frame shifts. After PCR amplification of the eGFP gene, primers were removed using PCR Purification Kit (QIAquick PCR Purification Kit, Qiagen, Hilden, Germany). The PCR amplicons were digested with BamHI and EcoRI and purified by running on an agarose gel followed by gel-extraction (QIAquick Gel Extraction Kit, Qiagen). Each frame-shifted gene encoding eGFP was introduced to pRSET B and pRSET-TriEX at the BamHI/EcoRI site. JM109(DE3) were transformed with the plasmids constructed, and fluorescence intensities were checked after overnight incubation at 37 °C. The digestion of the amplicons was performed prior to removing the PCR template plasmid, from which a trace amount of B frame gene encoding eGFP can be formed. Hence, a small amount of the B frame gene can be included in the A frame and C frame genes, which is a cause of pseudo-positive fluorescent colonies. The plates were illuminated with Transilluminator (TFML-26, UVP, LLC, Upland, CA, USA) equipped with Visi-Blue Plate (with Orange Cover) and imaged with a digital camera (PowerShot G11, Canon, Tokyo, Japan). The two pictures in Figure 3a,b were taken with identical exposure conditions. The exposure time was 4 s and the f-number was f/4.

Expression cloning was performed for the small jellyfish *Scolionema suvaense*. Total RNA of a single specimen was extracted with TRIzol reagent with the protocol supplied by the manufacturer. The cDNA was synthesized from the total RNA with SuperScript Double-Stranded cDNA Synthesis Kit (11917-010, Invitrogen, Carlsbad, CA, USA). The synthesized cDNA was phosphorylated using T4 Polynucleotide Kinase. pRSET-TriEX was digested with SmaI and dephosphorylated with Antarctic Phosphatase (New England Biolabs Inc.). The phosphorylated cDNA and linear dephosphorylated pRSET-TriEX were ligated and subjected to JM109(DE3) transformation. The transformants were plated onto an LB Amp plate. The fluorescence intensity of the colonies was checked after overnight incubation (18 h) at 37 °C. A single fluorescent colony on the plate was inoculated to 2 mL of an LB Amp medium and incubated overnight at 37 °C. The plasmid was prepared and sequenced using T7 promoter and T7 terminator reverse primers.

The CDS of ScSuFP was determined from the sequence data and amplified by PCR using forward primer, BamHI-C-ScSuFP-F (CGG GAT CCC ATG GAA GGC GGA ATG AAG C), and reverse primer, EcoRI-ScSuFP-R (CGG AAT TCT CAC AAT ATC GAA GTG ATC GGT TC). The PCR product was subcloned to empty pRSET B at BamHI /EcoRI site for the construction of pRSET-ScSuFP. 

For absorption and fluorescence spectrum analysis, ScSuFP with His-tag was expressed and purified. For protein expression, JM109(DE3) were transformed with pRSET-ScSuFP and plated onto an LB Amp plate. A colony was incubated in the LB Amp medium for 6 h at 37 °C, then incubated overnight at 20 °C. After that, expression was induced with 0.1 mM IPTG and incubated for 24 h at 20 °C. The *E. coli* cells were collected by centrifugation (4130× *g*, 15 min, 4 °C). The pellet was resuspended in phosphate buffered saline (PBS) and sonicated to homogenize it. Cell debris was separated from supernatant by centrifugation (7590× *g*, 10 min, 4 °C). The supernatant containing ScSuFP was purified using affinity chromatography with Ni-NTA Agarose (Qiagen), and the buffer was exchanged with Desalting column (PD-10, GE Healthcare UK Ltd., Little Chalfont, UK). Spectrum analysis of ScSuFP was performed in a phosphate buffer (10 mM, pH 7.0) supplemented with 150 mM NaCl. Absorption was measured with a spectrometer (Cary 100 UV-Visible Spectrophotometer, Agilent Technologies, Santa Clara, CA, USA), and fluorescence was measured with a spectrofluorometer (FluoroMax-3, HORIBA Jobin Yvon, Longjumeau, France). Fluorescence quantum yield was calculated using coumarin 153 (Fluorescence quantum yield in ethanol = 0.544) as a reference [33]. To determine the extinction coefficient, concentration of properly folded ScSuFP was estimated with an alkali-denatured method, using 0.1 M NaOH as a denaturant [27]. The reference extinction coefficient value of 44,000 M^−1^·cm^−1^ at 447 nm was used for the fully denatured chromophore. The concentration of whole ScSuFP was estimated with a theoretical extinction coefficient at 280 nm, which was calculated to be 31,860 M^−1^·cm^−1^ from the amino acid sequence [28]. In this calculation, we assumed that there is no intramolecular cysteine S–S bonding. 

Pseudo-native SDS PAGE analysis was performed on 15% polyacrylamide gel (0.375 M Tris HCl, pH 8.8, 0.1% SDS). Samples were loaded on the gel in a buffer containing 0.0625 M Tris HCl, pH 6.8, 1% SDS, 10% glycerol without boiling. Molecular weight standards were determined with a marker (XL-Ladder Broad, APRO Life Science Institute, Inc., Naruto, Japan). The photograph of the gel was taken with the same equipment as described above for the photographs of the plates.

The accession numbers of pRSET-TriEx and ScSuFP are LC363502 and LC361450, respectively.

These experiments have been approved by the Safety Committee for Gene Recombination Experiments of Yamagata University (Approval Number (Approval date): 22-47 (24 February 2011), 23-47(9 December 2011), 24-19 (8 June 2012), 24-31(26 November 2012), 25-23 (17 May 2013), 25-38 (12 November 2013), 26-31 (14 May 2014), 26-46 (20 November 2014), 27-30 (5 March 2015), 27-36 (1 May 2015), 27-42 (28 May 2015), 27-55 (4 December 2015), 28-30 (30 June 2016), 28-43 (24 November 2016), 28-60 (17 February 2017), 29-31 (25 May 2017), 29-36 (25 August 2017), 29-45 (21 November 2017)).
